# The endoplasmic reticulum membrane protein Sec62 as potential therapeutic target in *SEC62* overexpressing tumors

**DOI:** 10.3389/fphys.2022.1014271

**Published:** 2022-10-03

**Authors:** Julia S. M. Zimmermann, Johannes Linxweiler, Julia C. Radosa, Maximilian Linxweiler, Richard Zimmermann

**Affiliations:** ^1^ Department of Gynecology, Obstetrics and Reproductive Medicine, Saarland University, Homburg, Germany; ^2^ Department of Urology and Pediatric Urology, Saarland University, Homburg, Germany; ^3^ Department of Otorhinolaryngology, Head and Neck Surgery, Saarland University, Homburg, Germany; ^4^ Competence Center for Molecular Medicine, Saarland University, Homburg, Germany

**Keywords:** cellular calcium homeostasis, endoplasmic reticulum, endoplasmic reticulum-phagy, hallmarks of cancer, prodrug mipsagargin/G202, protein biogenesis, Sec62 protein, tumor driver gene SEC62

## Abstract

The human *SEC62* gene is located on chromosome 3q, was characterized as a tumor driver gene and is found to be overexpressed in an ever-growing number of tumors, particularly those with 3q26 amplification. Where analyzed, *SEC62* overexpression was associated with poor prognosis. Sec62 protein is a membrane protein of the endoplasmic reticulum (ER) and has functions in endoplasmic reticulum protein import, endoplasmic reticulum-phagy and -in cooperation with the cytosolic protein calmodulin- the maintenance of cellular calcium homeostasis. Various human tumors show *SEC62* overexpression in immunohistochemistry and corresponding cell lines confirm this phenomenon in western blots and immunofluorescence. Furthermore, these tumor cells are characterized by increased stress tolerance and migratory as well as invasive potential, three hallmarks of cancer cells. Strikingly, plasmid-driven overexpression of *SEC62* in non-*SEC62* overexpressing cells introduces the same three hallmarks of cancer into the transfected cells. Depletion of Sec62 from either type of *SEC62* overexpressing tumor cells by treatment with *SEC62*-targeting siRNAs leads to reduced stress tolerance and reduced migratory as well as invasive potential. Where tested, treatment of *SEC62* overexpressing tumor cells with the small molecule/calmodulin antagonist trifluoperazine (TFP) phenocopied the effect of *SEC62*-targeting siRNAs. Recently, first phase II clinical trials with the prodrug mipsagargin/G202, which targets cellular calcium homeostasis in prostate cells as well as neovascular tissue in various tumors were started. According to experiments with tumor cell lines, however, *SEC62* overexpressing tumor cells may be less responsive or resistant against such treatment. Therefore, murine tumor models for tumor growth or metastasis were evaluated with respect to their responsiveness to treatment with a mipsagargin analog (thapsigargin), or trifluoperazine, which had previously been in clinical use for the treatment of schizophrenia, or with the combination of both drugs. So far, no additive effect of the two drugs was observed but trifluoperazine had an inhibitory effect on tumor growth and metastatic potential in the models. Here, we review the state of affairs.

## Introduction

**FIGURE 1:**
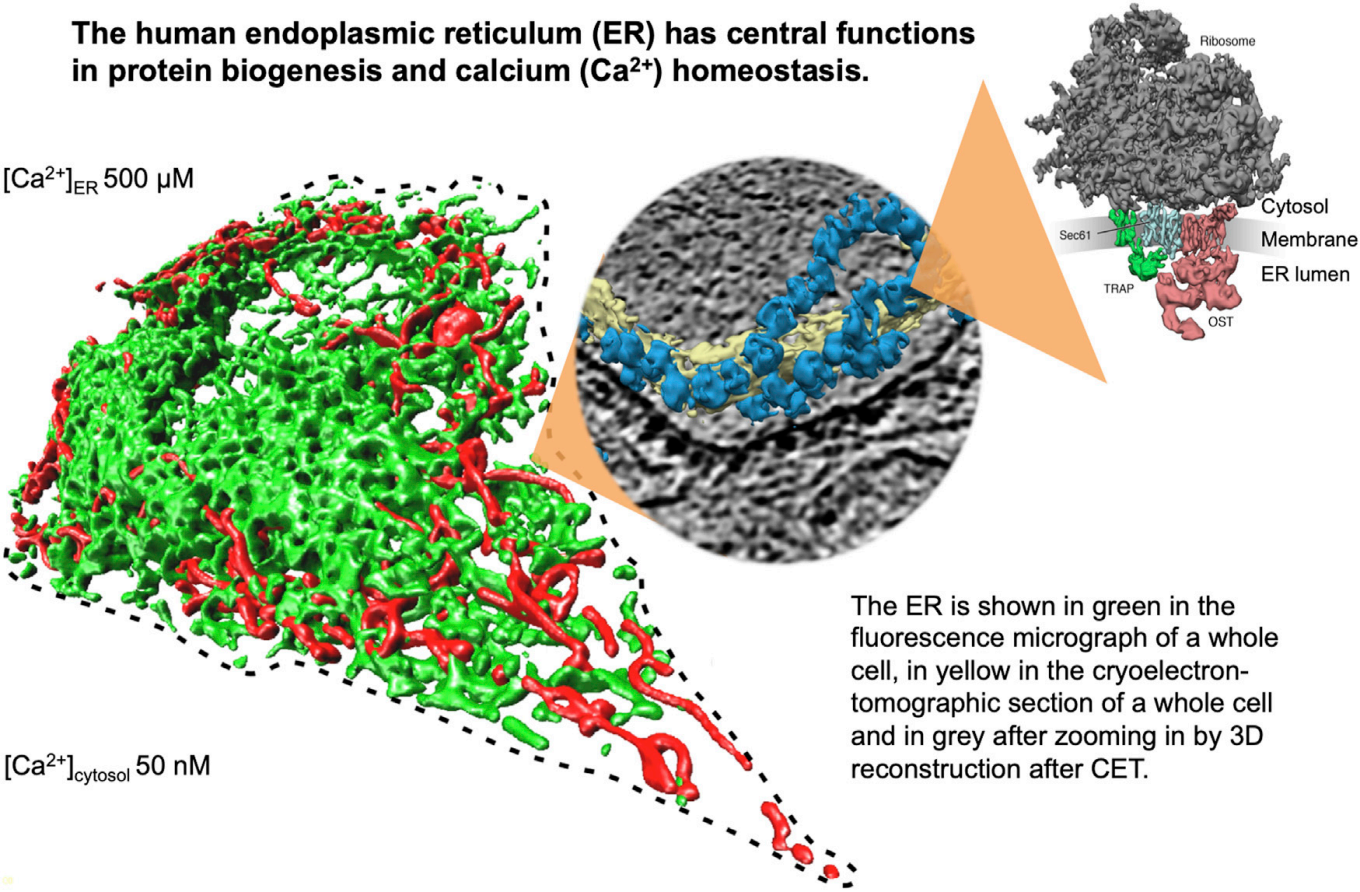
The endoplasmic reticulum (ER) of nucleated human cells has major functions in cellular calcium homeostasis, signal transduction and protein biogenesis. Going from left to right, it is shown here after fluorescence microscopy following expression of a GFP-targeted ER protein and a RFP-targeted mitochondrial protein, after cryoelectron tomography of a HeLa cell, and after 3D reconstruction of the human Sec61 complex together with its interaction partners ribosome, TRAP and oligosaccharyltransferase (Pfeffer et al., 2015). The Figure and Figure legend were adapted from Sicking et al. (2021).

**FIGURE 2:**
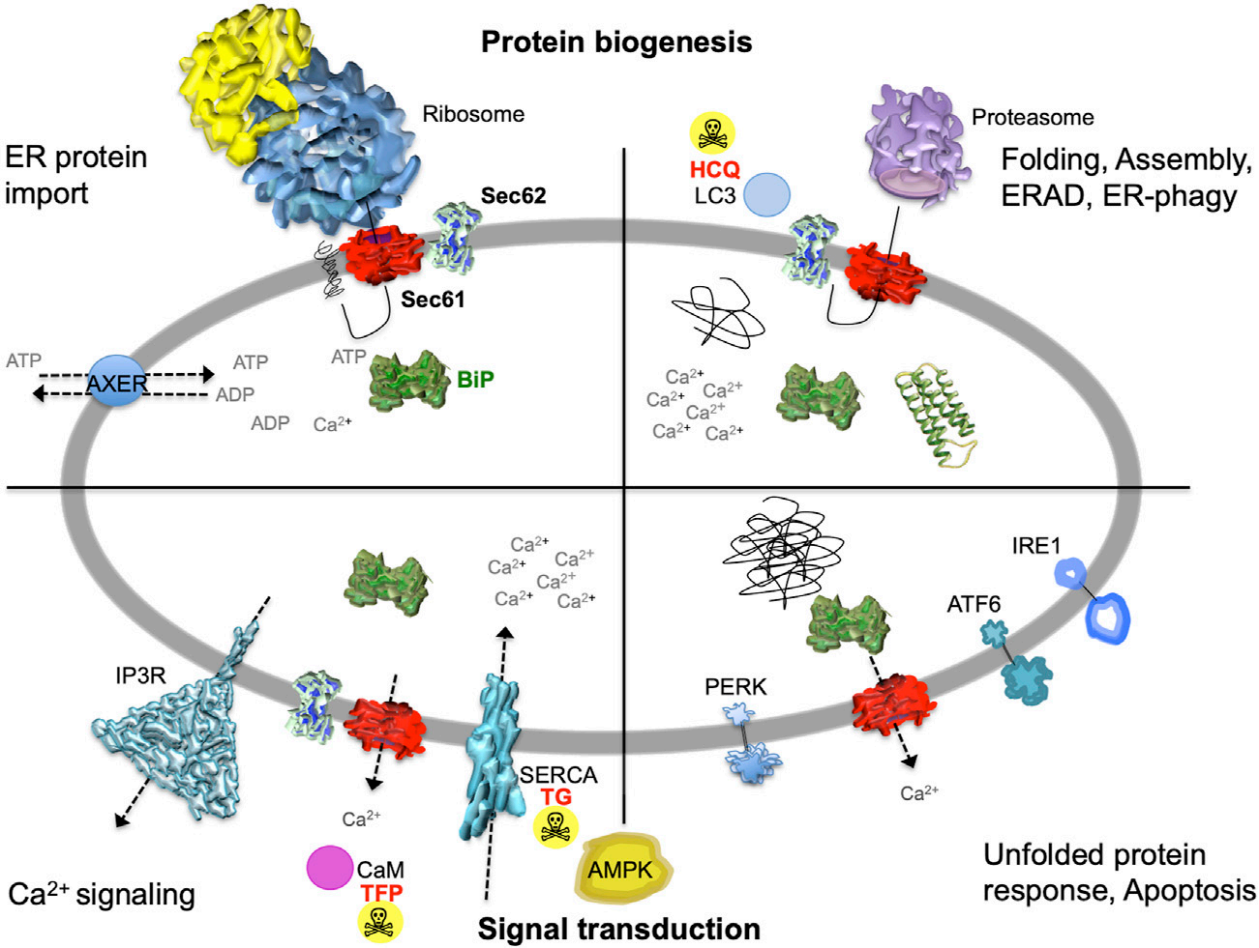
The heterotrimeric Sec61 complex, Sec62, IP3R and SERCA in the ER membrane are major players in protein biogenesis and Ca^2+^ -dependent signal transduction at the ER. These proteins are shown here in a schematic cross section of the human ER together with additional proteins and the various functions of the human ER. The non-annotated structures refer to a not yet-folded polypeptide, a natively folded protein, and an aggregate of non-native polypeptides, respectively. Although connected to the UPR, another level of complexity that is related to energy homeostasis of the ER lumen, particularly the ATP/ADP exchanger in the ER membrane (AXER/SLC35B1) and the ER to cytosol low energy signal transduction pathway, which involves cytosolic AMPK as well as Ca^2+^ efflux from the ER *via* the Sec61 channel, is not covered in this manuscript for the sake of clarity (Klein et al., 2018; Yong et al., 2019; Zimmermann and Lang, 2020; Schwarzbaum et al., 2022). AMPK, AMP-activated protein kinase; CaM, calmodulin; HCQ, hydroxychloroquine (autophagy inhibitor at the level of lysosomes); IP3R, IP3-receptor; SERCA, sarcoplasmic/endoplasmic reticulum Ca^2+^ ATPase; TFP, trifluoperazine (CaM antagonist); TG, thapsigargin (SERCA inhibitor). The Figure and Figure legend were adapted from Melnyk et al. (2022).

The endoplasmic reticulum (ER) of nucleated cells forms a vast membrane network, which extends from the nuclear envelope to the cell periphery (Figure 1, left) (reviewed by Lang et al., 2017; Lang et al., 2019; Molinari, 2020; Sicking et al., 2021; Walter and Ron, 2011). Briefly summarizing these reviews, the mammalian ER has major functions in gene expression, cellular calcium homeostasis and signal transduction (Figure 2). About thirty percent of the cellular proteome is synthesized by ER-bound ribosomes, which amounts to approximately ten thousand different proteins (Figure 1, center and right). Most of these proteins are either integrated into the ER membrane or imported into the ER lumen, as mediated by signal peptides or signal peptide-equivalent transmembrane helices in the precursor polypeptides. In addition, a complex transport machinery comprising about one hundred proteins in the cytosol, the ER membrane and the ER lumen is involved in these biosynthetic processes (Figure 2, upper left section). In the ER, the proteins are folded and assembled and non-ER proteins, such as plasma membrane or secretory proteins, are transported to the cell surface by vesicular transport. In the case of misfolding or misassembly of a certain protein due to mutation or of various proteins due to environmental conditions (such as low energy), ER-associated protein degradation (ERAD) kicks in or entire sections of the ER are destroyed by autophagy (ER-phagy) (Figure 2, upper right section). When these destructive mechanism fail in resolving the situation, signal transduction mechanisms are activated, which increase the capacity for folding and the destructive mechanisms in the affected cells, termed unfolded protein response (UPR), or initiate programmed cell death/apoptosis in order to save the organ or organism (Figure 2, lower right section). Besides these stress-related signal transduction processes, the ER plays a major role in Ca^2+^-mediated signal transduction that is closely related to cellular calcium homeostasis (Figure 2, lower left section). In a resting cell, there is an almost one thousand fold Ca^2+^ gradient between ER and the cytosol (Figure 1). However, upon stimulation of the cell by e.g. a hormone on the cell surface, the second messenger IP3 is produced near the plasma membrane and diffuses through the cytosol to the ER, where it binds to the IP3-receptor (IP3R). Activation of the IP3R causes massive efflux of Ca^2+^ from the ER and activates various signaling pathways, including increased expression of certain genes. When the cell stimulation subsides, the cell returns to the resting state and a Ca^2+^ pump in the ER membrane (termed sarcoplasmic/endoplasmic reticulum calcium ATPase/SERCA) regenerates the steep Ca^2+^ gradient between ER and cytosol. In addition, SERCA is constantly busy with pumping back Ca^2+^ into the ER lumen that had passively leaked from the ER. Recent years characterized the major ER protein import channel, the Sec61 channel, as one of the Ca^2+^ leak channels of the ER membrane in all nucleated cells. The Sec61 channel is associated with the ER membrane protein Sec62, which has functions in ER protein import, ER-phagy and keeping passive ER Ca^2+^ efflux *via* the Sec61 channel at bay (Figure 2).

**FIGURE 3:**
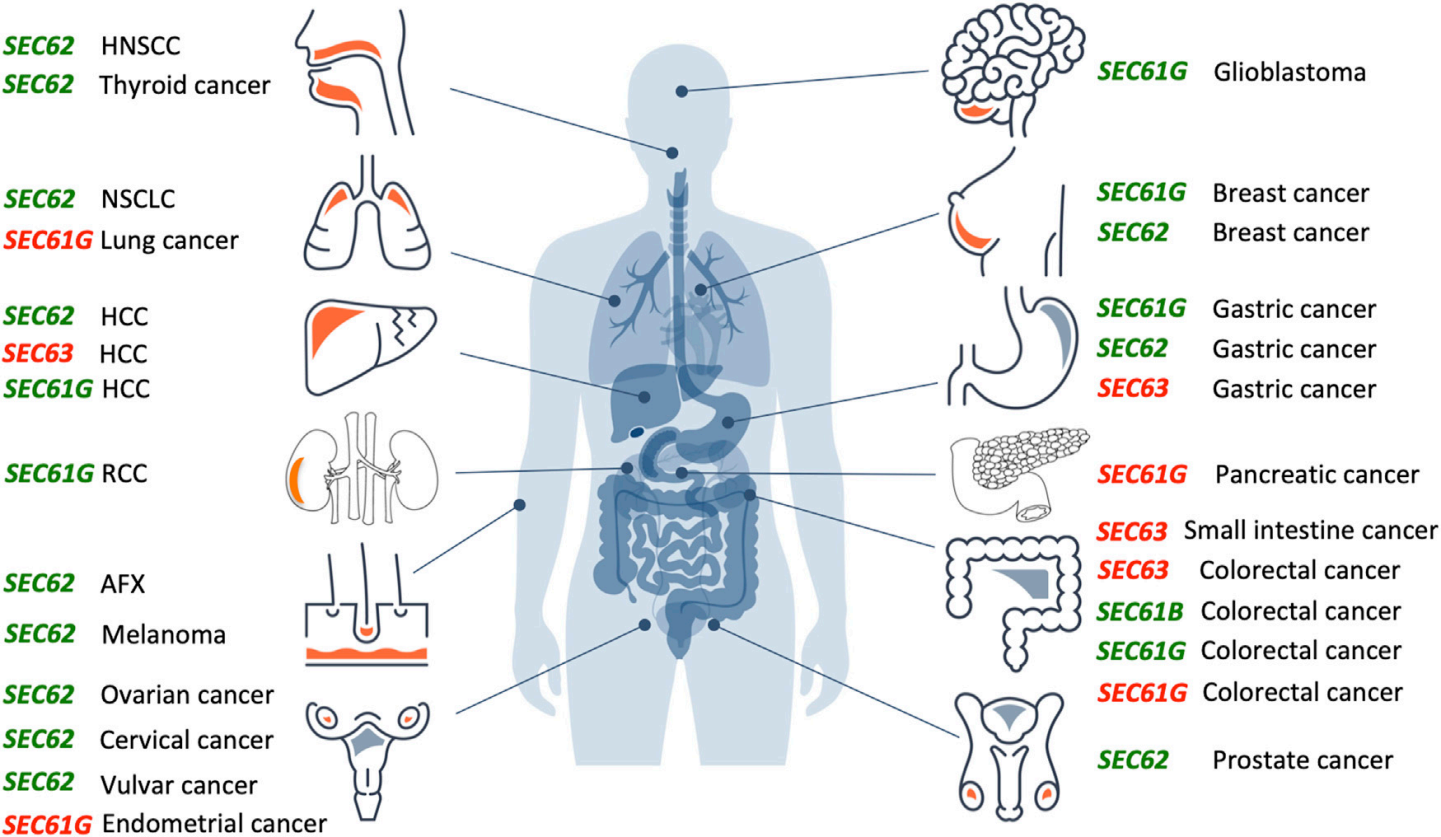
Overproduction or mutation of *SEC61*, *SEC62* and *SEC63* genes are associated with various human tumor diseases. Overview of genetic changes and altered expression of *SEC61*, *SEC62*, and *SEC63* gene in human cancer entities segregated by the tissue of origin (from top left to bottom right: head and neck, lung, liver, kidneys, skin, female genital tract, brain, breast, stomach, pancreas, intestine, male genital tract). Green colored genes symbolize functional gain by overexpression and amplification, red colored genes symbolize functional loss by low expression, deletion, or mutation. AFX, atypical fibroxanthoma; HCC, hepatocellular carcinoma; HNSCC, head and neck squamous cell carcinoma; NSCLC, non-small cell lung cancer; RCC, renal cell carcinoma.The Figure and Figure legend were adapted from Sicking et al. (2021).

**FIGURE 4:**
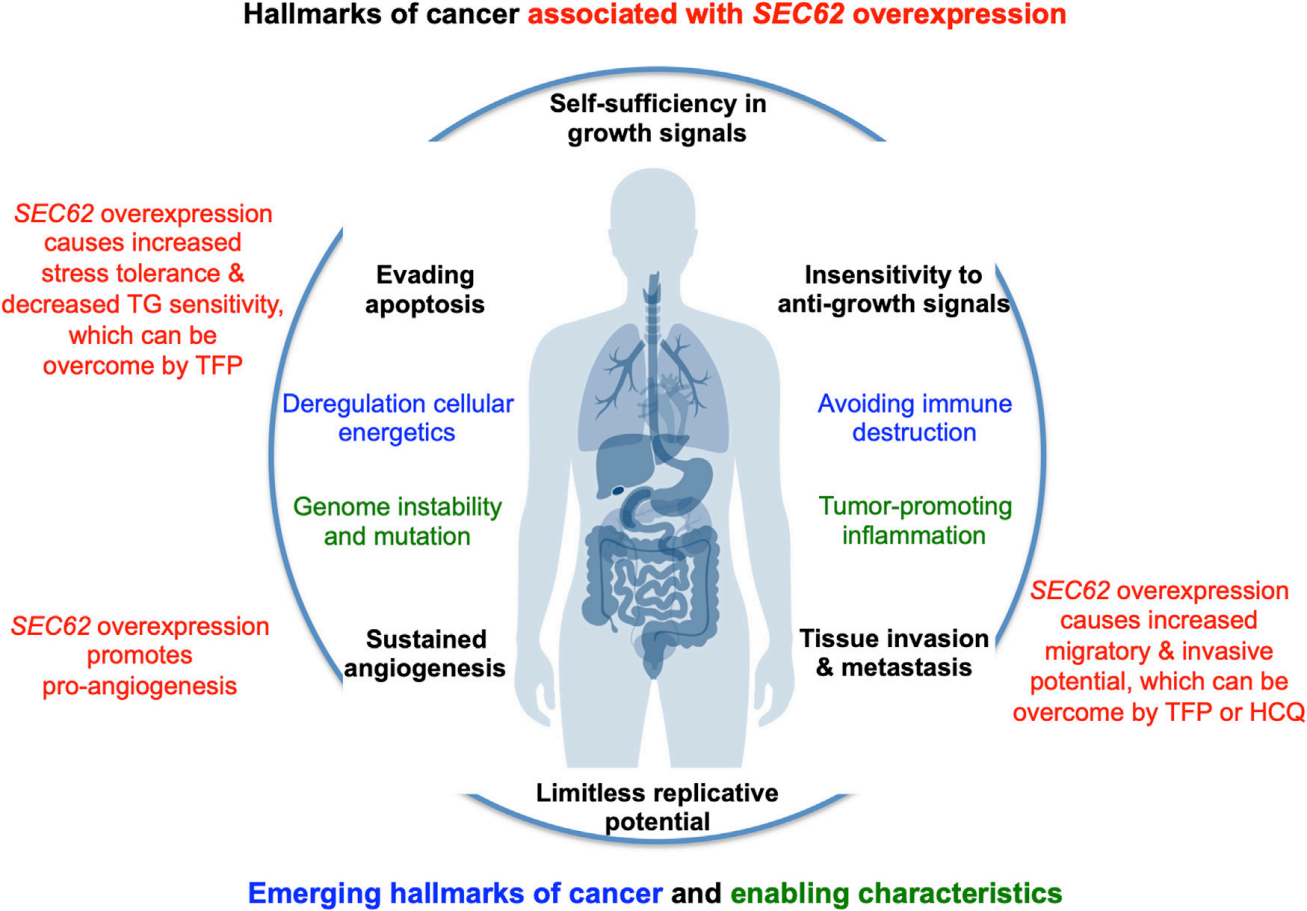
*SEC62* overexpressing tumor cells are characterized by increased stress tolerance and increased migratory and invasive potential, two hallmarks of cancer. The hallmarks of cancer and enabling characteristics were described in seminal papers by Hanahan and Weinberg (Hanahan and Weinberg, 2000; Hanahan and Weinberg, 2011). The red texts refer to the hallmarks that are linked to *SEC62* overexpressing cancer cells (Greiner et al., 2011a; Greiner et al., 2011b; Linxweiler et al., 2013).

In the year 2006, a first report associated the overexpression of the *SEC62* gene, which is also termed *TLOC1* and located at chromosome 3q26, with prostate cancer (Jung et al., 2006) (Figure 3). Subsequent studies identified *SEC62* overexpression as a phenomenon associated with prostate cancer progression in patients and, in prostate cancer cells, as the reason for increased ER stress tolerance as well as increased migratory and invasive potential (Greiner et al., 2011a; Greiner et al., 2011b). Thus, the latter observations associated *SEC62* overexpression with two hallmarks of cancer (Hanahan and Weinberg, 2000; Hanahan and Weinberg, 2011; Hanahan, 2022) (Figure 4). Therefore, the Sec62 protein was suggested as potential diagnostic marker as well as therapeutic target in prostate cancer (Greiner et al., 2011a). Subsequently, similar observations were made for non-small cell lung cancer (NSCLC) and thyroid cancer (Linxweiler et al., 2012; Linxweiler et al., 2014) and *SEC62* overexpression was linked to poor prognosis in NSCLC (Linxweiler et al., 2013). Furthermore, the latter work characterized a clinically tested small molecule and Calmodulin-antagonist (trifluoperazine/TFP) as being able to suppress the effects of *SEC62* overexpression on ER stress tolerance and migratory potential of tumor cells. Also in 2013, the *SEC62/TLOC1* gene was characterized as tumor driver gene (Hagerstrand et al., 2013). Next, head and neck squamous cell carcinomas (HNSCC) and cervical cancer of unknown primary (CUP) were added to the list of *SEC62* overexpressing tumors (Wemmert et al., 2016; Bochen et al., 2017) as were dysplastic cervical lesions (Linxweiler et al., 2016), atypical fibroxanthoma and melanoma (Müller et al., 2019; Müller et al., 2021), mammary carcinoma/invasive ductal breast cancer, vulvar cancer/vulvar intraepithelial neoplasia, cervical cancer/cervical intraepithelial neoplasia (Takacs et al., 2019a; Takacs et al., 2019b; Takacs et al., 2019c) and gastric cancer (Su et al., 2022). Therefore, it appears that Sec62 may be a diagnostic and prognostic marker as well as a therapeutic target for various tumors, which warranted murine tumor models as the next logical step (Linxweiler et al., 2017). In light of the exciting developments in the field of targeted SERCA-inhibiting prodrugs, developed by John T. Isaacs, Sören B. Christensen and colleagues in the fight against prostate cancer as well as neovascular tissue in various tumors (Treiman et al., 1998; Denmeade et al., 2003, 2012; Doan et al., 2015; Mahalingam et al., 2016), we review the current state of the studies on *SEC62* overexpressing tumors and discuss where, in our opinion, the field should go from here.

## Summary of results on mammalian Sec62 protein

### Functions of the endoplasmic reticulum membrane protein Sec62

**FIGURE 5:**
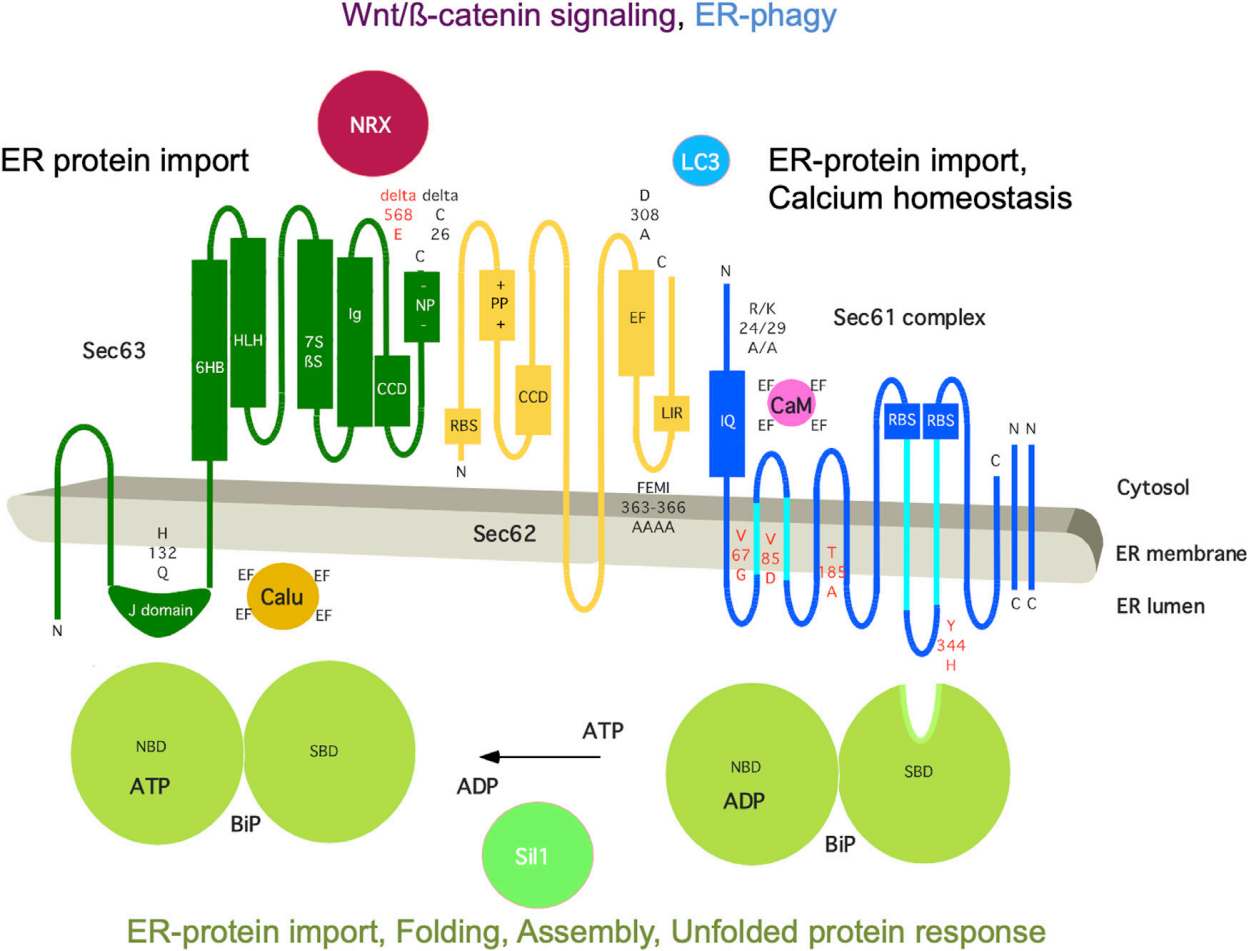
The human ER membrane protein Sec62 is part of a dynamic protein network. Topology and functionally relevant domains of the heterotrimeric Sec61 complex and its allosteric effectors BiP, Sec62 and Sec63 are shown (Mayer et al., 2000; Tyedmers et al., 2000). The cytosolic loops six and eight of the α-subunit of the Sec61 complex comprise a binding site for ribosomes (termed RBS) and ER-lumenal loop 7 a binding site for BiP (Schäuble et al., 2012; Cheng et al., 2015); the N-terminus of the same subunit contains an IQ-motif for binding of Ca^2+^-calmodulin (CaM) (Erdmann et al., 2011). The cofactors of BiP, Sec63 and Sil1, are also shown, as are additional interaction partners of Sec62 (LC3) and Sec63 (nucleoredoxin/NRX, calumenin/Calu) (Tyedmers et al., 2005; Müller et al., 2011; Fumagalli et al., 2016). Disease-associated mutations of Sec61α and Sec63 are indicated in red (amino acid residues are given in single letter code) (reviewed by Sicking et al., 2021), as are rationally designed mutations in black, such as Sec61α^R24A/K29A^ (Erdmann et al., 2011), Sec62^D308A^ (Linxweiler et al., 2013), Sec62^FEMI363-366AAAA^ (Fumagalli et al., 2016), and Sec63^H132Q^ as well as Sec63^deltaC26^ (Müller at al., 2010; Haßdenteufel et al., 2018; Schorr et al., 2020). C, C-terminus; CCD, coiled-coil domain for protein interaction; EF, EF hand; IQ, IQ-motif for CaM binding; LIR, LIR-motif for LC3 binding; N, N-terminus; NBD, nucleotide binding domain; -NP-, patch with negatively charged amino acid residues; +PP+, patch with positively charged amino acid residues; RBS, ribosome binding site; SBD, substrate binding site. The Figure and Figure legend were adapted from Sicking et al. (2021).

The mammalian Sec62 protein is in the center of a dynamic protein network (Figure 5). It is present in the ER membrane, contains two transmembrane helices, a short ER-lumenal loop plus two large cytosolic domains, forms a heterodimeric complex with the Sec63 protein and transiently associates with the heterotrimeric Sec61 complex (Daimon et al., 1997; Mayer et al., 2000; Tyedmers et al., 2000). These interactions involve a positively charged patch of amino acid residues in the N-terminal domain of Sec62 plus a negatively charged cluster at the C-terminus of Sec63 and the C-terminal domain of Sec62 (including two predicted EF hands) plus the N-terminus of Sec61α, respectively (Müller et al., 2010; Linxweiler et al., 2013). Notably, the Sec62/Sec63 interaction is enhanced by CK2-mediated phosphorylation within the negatively charged patch of Sec63 (Ampofo et al., 2013) and the Sec62/Sec61 interaction is sensitive to Ca^2+^, likely involving the EF hands in Sec62 (Linxweiler et al., 2013). Cytosolic interaction partners of Sec62 are Ca^2+^ (the two predicted EF hands), ribosomes and LC3 and involve at the level of Sec62 the putative EF hands, a ribosome binding site (RBS) and a LIR-motif within the more C-terminal EF hand, which are relevant for cellular calcium homeostasis, ER protein import and ER-phagy, respectively (Müller et al., 2010; Linxweiler et al., 2013; Fumagalli et al., 2016). The α-subunit of the Sec61 complex contains ten transmembrane helices, interacts with ribosomes *via* two RBS and contains an IQ-motif at its cytosolic N-terminus, which provides a binding site for the cytosolic EF hand protein calmodulin (CaM) (Kalies et al., 1994; Erdmann et al., 2011; Cheng et al., 2015). The Sec63 protein contains three transmembrane helices and two domains in cytosol and ER lumen, respectively, and comprises a binding site for nucleoredoxin (NRX) near the cytosolic C-terminus and an ER-lumenal J-domain for the interactions with the chaperone BiP plus the EF hand protein calumenin (Tyedmers et al., 2005; Weitzmann et al., 2007; Dudek et al., 2009; Müller et al., 2011; Melnyk et al., 2022). The roles of the J-domain of Sec63 are to recruit BiP to the Sec62/Sec63 complex, to stimulate the ATPase activity of BiP and, thereby, to allow activated BiP to bind a substrate, such as an oligopeptide within ER-lumenal loop seven of Sec61α (Weitzmann et al., 2007; Schäuble et al., 2012). Notably, BiP is present in the ER lumen at millimolar concentration, comprises a nucleotide-binding domain (NBD) plus a substrate binding domain (SBD) as well as multiple low affinity Ca^2+^ binding sites, and depends on ATP as well as a high ER-lumenal Ca^2+^ concentration for its activity (Melnyk et al., 2022). Sil1 is one of the nucleotide exchange factors for BiP, Grp170 is another one (Weitzmann et al., 2006). BiP also interacts with the ER-lumenal domains of ATF6, IRE1 and PERK and, thus, is connected to the UPR (Walter and Ron, 2011). In addition, IRE1α interacts with Sec61 and, thus, directly links ER protein import and Ca^2+^ leakage with UPR (Sundaram et al., 2017; Li et al., 2020). Interestingly, NRX interacts with protein Dishevelled 1 (Dvl1) and, thus, is linked to the Wnt signaling pathway and cadherin-controlled cell adhesion (Nelson and Nusse, 2004; Nusse and Clevers, 2017). It was proposed that oxidative stress simultaneously inhibits NRX/Dvl1 interaction and stimulates NRX/Sec63 interaction, thereby activating Wnt/β-catenin signaling as well as affecting β-catenin/cadherin-controlled cell adhesion, which both are linked to cell migration and various cancers (Müller et al., 2011).

The Sec61 complex in the membrane of the ER provides the major entry point for precursor polypeptides with either N-terminal signal peptides or equivalent transmembrane helices into the ER lumen and membrane, respectively (Figures 1, 5) (reviewed by Lang et al., 2017; Lang et al., 2019). In the early phase of co- and posttranslational membrane translocation or integration, the signal peptides or transmembrane helices of precursor polypeptides are targeted to the Sec61 complex by various pathways. Next, binding of the precursor proteins to the closed Sec61 complex triggers opening of an aqueous polypeptide-conducting channel, which is formed by Sec61α (Pfeffer et al., 2015). This occurs spontaneously after binding of precursors with strong signal peptides to the channel or with additional support from auxiliary components, such as TRAP (Figure 1, right) or Sec62/Sec63 plus BiP (Figure 5). Thus, while the Sec61-complex mediates import of most precursor polypeptides into the ER, the Sec61-associated Sec62/Sec63 heterodimer supports ER protein import in a precursor-specific manner, typically in cooperation with the ER-lumenal Hsp70-type molecular chaperone BiP. Analyses of protein transport in human and murine cells showed a client-specific role of Sec62 in ER protein import, such as the posttranslational ER import of presecretory proteins with a content of less than 100 amino acid residues (Lakkaraju et al., 2012; Lang et al., 2012; Haßdenteufel et al., 2018; Haßdenteufel et al., 2019). However, these small precursor proteins do not represent the complete picture of Sec62 substrates, since the human Sec62/Sec63 complex was also found to be involved in cotranslational import of certain large precursor polypeptides, such as prion protein. Notably, the mammalian Sec62 protein is able to interact with the ribosome and to recruit, in collaboration with Sec63, BiP to the Sec61 channel. The latter interaction facilitates Sec61 channel opening for large precursors with weak signal peptides (Ziska et al., 2019; Schorr et al., 2020), the idea being that binding of BiP to loop seven of Sec61α provides binding energy for shifting the dynamic equilibrium of the Sec61 channel to the open state (Schäuble et al., 2012). The BiP binding site in Sec61α was characterized as a di-tyrosine motif–containing mini-helix in ER-lumenal loop 7 (Figure 5). Interestingly, homozygous mutation of tyrosine 344 to histidine in this loop seven is linked to Diabetes mellitus in mice and compromises co- and post-translational ER import of Sec63-plus BiP-dependent precursor polypeptides when introduced into HeLa cells. Furthermore, BiP can bind to incoming precursor polypeptides and act on these as a molecular ratchet, which guarantees unidirectional transport (Tyedmers et al., 2003). Typical for an Hsp70, both BiP activities involve ATP, Ca^2+^, J-domain-proteins or Hsp40-type co-chaperones, such as Sec63, and nucleotide exchange factors, such as Sil1 (Melnyk et al., 2022). Following the same principles and interactions, BiP also plays a central role in folding and assembly of newly-imported polypeptides, such as immunoglobulins in the plasma cells of the immune system (reviewed by Melnyk et al., 2022), and supports efficient Sec61 channel closing to preserve Ca^2+^ homeostasis (Schäuble et al., 2012), which will be discussed next. In addition, BiP is a key player in various Ca^2+^–dependent and–independent signal transduction pathways, which report on ER energy- and protein-homeostasis (see below).

The mammalian ER also represents the major Ca^2+^ storage compartment in nucleated cells and allows the controlled release of Ca^2+^ from the ER. Interestingly, the open polypeptide-conducting Sec61 channel allows the passive passage of Ca^2+^ from the ER and, therefore, needs to be closely controlled (Wirth et al., 2003; Lang et al., 2011). Sec61 channel closing either occurs spontaneously or is facilitated by allosteric effectors, such as the ER-lumenal BiP and/or the cytosolic Ca^2+^-calmodulin (Ca^2+^-CaM) (Erdmann et al., 2011; Schäuble et al., 2012). Originally, single-channel recordings from planar lipid bilayers characterized the Sec61 complex as a highly dynamic aqueous channel that is transiently opened by signal peptides and permeable to Ca^2+^ after completion of protein import (Wirth et al., 2003; Erdmann et al., 2011). The same biophysical approach showed that the Sec61 channel closure can be induced by binding of BiP or Ca^2+^-CaM (Erdmann et al., 2011). Next, the fact that BiP is involved in closing the Sec61 channel was confirmed at the cellular level by combination of siRNA-mediated gene silencing or pharmacological manipulation and live cell Ca^2+^ imaging (Schäuble et al., 2012). In addition, cytosolic Ca^2+^-CaM was shown under similar conditions to contribute to Sec61 channel closing *via* an unrelated mechanism after Ca^2+^ has started to leak from the ER (Erdmann et al., 2011). During the last 15 years, additional siRNA-mediated gene silencing and live cell Ca^2+^ imaging experiments identified the ER-lumenal J-domain proteins ERj3 and ERj6 as specific co-chaperones of BiP for channel closure and the putative EF hand- and Ca^2+^- binding protein Sec62 as a co-factor of CaM in Sec61 channel closing (Figures 2, 5) (Linxweiler et al., 2013; Schorr et al., 2015). The binding site of BiP was identified as the above-mentioned di-tyrosine motif–containing mini-helix within ER-lumenal loop seven of the Sec61α and was shown to be relevant to the described mechanisms by mutagenesis studies (Schäuble et al., 2012). Again, the idea is that binding of BiP to loop seven of Sec61α provides binding energy for shifting the dynamic equilibrium of the Sec61 channel to the closed state. In case of inefficient channel closure in intact cells, Ca^2+^ starts to leak from the ER into the cytosol and binds calmodulin in the ER vicinity, and Ca^2+^-CaM is recruited to the IQ-motif in the Sec61 α-subunit (Erdmann et al., 2011) (Figures 2, 5). Once more, the involved binding energy favors channel closure. Apparently, binding of Ca^2+^-CaM is supported by Sec62, which may bind Ca^2+^ because of its putative EF hands within its cytosolic C-terminal domain and lets go of the Sec61 α-subunit (Linxweiler et al., 2013). After the Sec61 channel is closed, and Ca^2+^ leakage subsides, SERCA pumps Ca^2+^ back into the ER, CaM and Sec62 return to the respective Ca^2+^-free forms, and the next protein import cycle can be initiated. When these mechanisms fail, however, the passive Ca^2+^ efflux from the ER may lower BiP activity and, therefore, cause protein misfolding, which may be reported *via* the UPR, and, eventually lead to apoptosis.

Recently, a function of the mammalian Sec62 protein beyond its house-keeping functions in ER protein import and calcium homeostasis was found and, in contrast to the latter two, is activated by demand, possibly by dephosphorylation of the C-terminal domain of Sec63 or by Ca^2+^ binding to the predicted EF hands of Sec62. Fumagalli et al. observed that monomeric Sec62 plays a crucial role in the recovery of human and murine cells from ER stress (Fumagalli et al., 2016). The term ER stress describes conditions of globally disturbed folding and assembly of proteins in the ER, which can be caused e.g. by reduced ER levels of ATP or Ca^2+^. Depending on the stress severity, the cell can either stimulate compensatory mechanisms such as the UPR or initiate apoptosis, which may involve Sec61 channel mediated Ca^2+^ efflux from the ER (Hara et al., 2014; Feliziani et al., 2020). During UPR, synthesis of the majority of proteins is inhibited while the production of ER chaperones such as BiP is drastically increased in order to facilitate the correct folding of ER-lumenal polypeptides. In the case that this rescue operation fails, misfolded proteins are degraded by the proteasome *via* ERAD or *via* ER-phagy (Pisoni and Molinari, 2016; Molinari, 2020). If the cell succeeds in coping with the ER stress, the expanded ER with its increased amount of ER-lumenal chaperones has to be brought back to a physiological size.

Therefore, small vesicles bearing ER-lumenal chaperones are pinched-off from the ER and engulfed by endolysosomes for degradation in an ER-phagy-related process, termed piecemeal micro-ER-phagy (Loi et al., 2019) (Figure 2). In this context, Sec62 was shown to bear a LIR-motif at its C-terminus functioning as a receptor for LC3 during recovery from ER stress e.g. as induced by the reversible SERCA inhibitor cyclopiazonic acid (Fumagalli et al., 2016). The interaction between the LIR-motif and LC3 is sensitive to mutation of the tetrapeptide _363_FEMI_366_ to tetraalanine (Figure 5). Thus, Sec62 plays an important, Sec61-and Sec63-independent, role during the compensation of ER stress, termed recovER-phagy. It remains to be seen if Sec62 also plays a general role in ER-phagy that is involved in fighting ER stress and how it is activated for its role in these processes. It would not come as a surprise if the interaction of IRE1α with Sec61, which links ER protein import and Ca^2+^ leakage with UPR, would play a role in one way or another (Sundaram et al., 2017; Li et al., 2020).

### *SEC61, SEC62* and *SEC63* gene overexpression or mutation associated with human tumor diseases

Over the past 15 years, increasing evidence suggests a relevant role of *SEC61*, *SEC62* and *SEC63* genes in the development and tumor cell biology of human malignancies (Figure 3). For the *SEC61B* gene altered expression was reported for colorectal cancer (reviewed by Fan et al., 2011). Beginning in 2006, for the *SEC61G* gene increased expression and gene amplification were reported for breast cancer, glioblastoma, gastric cancer, colorectal cancer, renal cancer and lung adenocarcinoma (Reis-Filho et al., 2006; Tsukamoto et al., 2008; Lu et al., 2009; Meng et al., 2021a; Chen et al., 2010; Xu et al., 2022). In glioblastoma multiforme, Liu et al. observed a significant correlation of high *SEC61G* expression with poor prognosis based on statistical analysis of data from the Cancer Genome Atlas cohort and the Chinese Glioma Genome Atlas cohort (Liu et al., 2019). Univariate and multivariate Cox proportional hazards regression verified *SEC61G* as an independent factor for prognosis and therapeutic outcome in these cohorts. Furthermore, mining of the COSMIC database identified in 2021 six mutations in the *SEC61G* gene that were associated with colorectal, endometrial, pancreatic and lung cancer, respectively, and linked to disturbed cellular calcium homeostasis due to altered Sec61 channel gating (Witham et al., 2021). In 2002, a first publication described frameshift mutations of *SEC63* due to microsatellite instability in 38% of gastric cancers and 49% of colorectal cancers (Mori et al., 2002). Similar results were reported in 2005 and 2013 (Eschrich et al., 2005; Schulmann et al., 2005; Casper et al., 2013; Casper et al., 2021), where microsatellite instability associated *SEC63* frameshift mutations were found in 56% of small-bowel cancers in patients with hereditary non-polyposis colorectal cancer (HNPCC) and in one case of hepatocellular carcinoma (HCC).

However, most extensive evidence for a causative role of a protein translocation component in the development and tumor cell biology of human cancer exists for the ER transmembrane protein Sec62. In 2006, a first study found *SEC62* copy number gains in 7 of 13 prostate cancer samples as well as elevated Sec62 protein levels in three prostate cancer cell lines (Jung et al., 2006). In the following years, amplification and overexpression of the *SEC62* gene were reported for various other cancer entities, including non-small cell lung cancer (NSCLC) (Greiner et al., 2011a; Linxweiler et al., 2012), thyroid cancer (Greiner et al., 2011a; Linxweiler et al., 2012), hepatocellular cancer (Weng et al., 2012; Du et al., 2019), ovarian cancer (Hagerstrand et al., 2013; Radosa et al., 2022), breast cancer (Hagerstrand et al., 2013; Takacs et al., 2019a), head and neck squamous cell carcinoma (HNSCC) (Wemmert et al., 2016; Bochen et al., 2017), cervical cancer (Takacs et al., 2019b), vulvar cancer (Takacs et al., 2019c), atypical fibroxanthoma (Müller et al., 2019), melanoma (Müller et al., 2021), gastric cancer (Su et al., 2022) and in larger prostate cancer patient cohorts (Greiner et al., 2011b). After screening the cBio portal for cancer genomics from over 72,000 cancer patients with 55 different tumor entities *SEC62* gene alterations were reported for 2,595 patients and represented gene amplifications in the majority of cases (Cerami et al., 2012; Gao et al., 2013; Sicking et al., 2021). Up to now, *SEC62* overexpression was linked to poor prognosis in prostate cancer (Jung et al., 2006), hepatocellular cancer (Weng et al., 2012; Du et al., 2019), NSCLC (Linxweiler et al., 2013), HNSCC (Wemmert et al., 2016; Bochen et al., 2017), breast cancer (Takacs et al., 2019a), melanoma (Müller et al., 2021), colorectal cancer (Liu et al., 2021), ovarian cancer (Radosa et al., 2022) and gastric cancer (Su et al., 2022).

### *SEC62* overexpressing tumor cells have increased stress tolerance and migratory as well as invasive potential

From a functional point of view only few studies addressed the specific functional impact of altered *SEC62* expression levels on cancer cell biology. A first step to uncover potential associations of *SEC62* overexpression with tumor cell biology were correlative analyses with clinical data. Thereby, Greiner et al. found an association of high *SEC62* expression level with higher Gleason Score in prostate cancer (Greiner et al., 2011b). In non-small cell lung cancer, high Sec62 levels correlated with the occurrence of lymph node metastases and tumor cell de-differentiation (Linxweiler et al., 2012). Similarly, an association of *SEC62* overexpression with lymphatic metastases was reported for head and neck squamous cell carcinoma (Bochen et al., 2017) as well as an association of *SEC62* overexpression with distant metastases in breast cancer (Takacs et al., 2019a). These results indicated a potential role of Sec62 in cancer metastasis, which was strengthened by several functional studies. As a second step, a significant inhibition of cancer cell migration by *SEC62* gene silencing was reported for prostate cancer cells (Greiner et al., 2011b), non-small cell lung cancer cells as well as thyroid carcinoma cells (Linxweiler et al., 2012), cervical cancer cells (Linxweiler et al., 2016), hepatocellular carcinoma cells (Du et al., 2019; Li et al., 2019) and head and neck squamous cell carcinoma cells (Bochen et al., 2017). Where tested, overproduction of a mutated Sec62, Sec62^D308A^ (Figure 5) that affected the more N-terminal EF hand had a similar effect on cell migration as *SEC62* silencing, i.e. an even dominant negative effect over Sec62 (Linxweiler et al., 2013). Strikingly, *SEC62* overexpression stimulated the migratory potential and stress tolerance of otherwise poorly migrating and poorly stress tolerant cells such as HEK293, HeLa, Huh-7 and FaDu cells (Greiner et al., 2011a; Linxweiler et al., 2013; Bochen et al., 2017; Du et al., 2019). Furthermore, an influence of *SEC62* expression level on stress tolerance of human cancer cells was suggested by several studies that reported a higher ER stress sensitivity induced by *SEC62* silencing, which was phenocopied by overproduction of Sec62^D308A^ or administration of CaM antagonists, such as TFP (Greiner et al., 2011b; Linxweiler et al., 2012; Linxweiler et al., 2016).

These findings raised the question of how *SEC62* overexpression causes increased stress tolerance and migratory and invasive potential of the respective cancer cells:

1) On first sight, the role of Sec62 in ER protein import may appear to be an unlikely candidate since the protein typically cooperates with Sec63 and simultaneous *SEC63* overexpression was not observed in *SEC62* overexpressing tumor cells (Greiner et al., 2011a). However, in our opinion it cannot entirely be dismissed since in some cases Sec62 was found to play a role in ER protein import without the involvement of Sec63 (Haßdenteufel et al., 2018; Lang et al., 2022). In this context it is tempting to speculate that the biogenesis of ADAM metalloproteinases, which are involved in proteolytic cleavage of extracellular matrix and cell adhesion proteins, as well as cadherins that play a direct role in cell adhesion, is limited by standard Sec62 levels. Therefore, it may be improved by increased levels of Sec62, thereby supporting cell migration. Indeed, the two α-secretases ADAM10 and ADAM17 were found to depend on Sec62 but not Sec63 in their ER import in HeLa cells (reviewed by Lang et al., 2022). As a caveat, however, loss of cadherin expression was found to promote tumorigenesis (Nelson and Nusse, 2004).
2) Therefore, the increased suppression of Ca^2+^ efflux *via* the Sec61 channel by Sec62 and the activated ER-phagy in *SEC62* overexpressing cancer cells may appear as the more likely reasons for the improved migratory potential and stress tolerance (Erdmann et al., 2011; Fumagalli et al., 2016; Linxweiler et al., 2016; Bergmann et al., 2017). Increased suppression of Ca^2+^ efflux *via* the Sec61 channel by increased levels of Sec62 may prevent apoptosis, which involves an elevated cytosolic Ca^2+^ concentration, in combination with an increased capacity for ER-phagy due to the excess of free Sec62 may allow the cells to defend themselves instantly and more efficiently against ER stress and, thereby, also to prevent apoptosis. As another caveat, however, the mutations of the *SEC61G* gene that were associated with colorectal, endometrial, pancreatic and lung cancer were found to cause increased Ca^2+^ efflux from the ER due to altered Sec61 channel gating (Witham et al., 2021).

Overall, we favor the idea that all these Sec62 functions may be responsible for the increased stress tolerance and migratory potential of the *SEC62* overexpressing cancer cells. Indeed, *SEC62* overexpression but not overproduction of the EF hand mutant variant Sec62^D308A^ allowed cells to tolerate higher levels of the irreversible SERCA inhibitor thapsigargin (TG) (Treiman et al., 1998; Lindner et al., 2020). Notably, however, cytosolic Ca^2+^ also plays a role in cell migration (Tsai et al., 2015), which has to be considered as a possible contributing factor, and the Sec63 interaction partner NXN may be relevant here by activating Wnt/β-catenin signaling or affecting β-catenin/cadherin-controlled cell adhesion (Nelson and Nusse, 2004; Müller et al., 2011; Nusse and Clevers, 2017). Furthermore, the recently reported observation that *SEC62* overexpression leads to overproduction of PAI-1 (coded by the *SERPINE1* gene) and TNFRSF11B and the putative resulting hypoxia-induced tube formation as well as stimulated Wnt/β-catenin signaling also have to be taken into future account (Meng et al., 2021b). Notably, it was suggested that it needs to be addressed if autophagy inhibitors such as bafilomycin A1, chloroquine (CQ) and hydroxychloroquine (HCQ) can cause decreased stress tolerance and/or decreased migratory as well as invasive potential (Bergmann et al., 2017). Alternatively, the overexpression of the recovER-phagy-deficient mutant variant Sec62^FEMI363-366AAAA^ should be tested as a potential cause for increased stress tolerance as well as increased migratory and invasive potential.

### Depletion of Sec62 from tumor cells or treatment of the cells with the drug trifluoperazine reduce stress tolerance and migratory as well as invasive potential

Taken together, these data strongly indicate a role of *SEC62* as a driver oncogene in various human cancers, which show a consistent association with poor prognosis, lymph node as well as distant metastasis and stress tolerance. This turns the Sec62 protein into an attractive target for anticancer therapy (Greiner et al., 2011a; Linxweiler et al., 2017). Since *SEC62*-targeting siRNAs are not a therapeutic option at present and Sec62 is not accessible to monoclonal antibodies due to its intracellular location, alternative strategies had to be developed to achieve at least a functional knock-down in *SEC62* overexpressing tumors.

Cell death triggered by unmitigated ER stress is seen as an important potential therapeutic strategy in cancer therapy (Denmeade et al., 2003; Lindner et al., 2020). Based on the role of Sec62 in the regulation of Ca^2+^ efflux through the Sec61 channel, CaM antagonists (e.g. TFP) were investigated as potential therapeutics at the level of cancer cell lines (Vandonselaar, et al., 1994; Linxweiler et al., 2013). Notably, TFP had previously been in clinical use for the treatment of schizophrenia (Marques et al., 2004). Interestingly, CaM antagonists such as TFP mimicked a functional Sec62 knockdown by stimulating Ca^2+^ efflux from the ER and inhibiting the migratory potential of cervix and prostate cancer cells, which was linked to inhibition of proliferation at lower doses of TG (Linxweiler et al., 2012; Linxweiler et al., 2016).

These findings on TFP have to be seen in light of the exciting developments in the field of SERCA-targeting prodrugs, developed by John T. Isaacs, Sören B. Christensen and colleagues, originally in the fight against prostate cancer as well as more recently directed at neovascular tissue in various tumors (Denmeade et al., 2003; Denmeade et al., 2012; Doan et al., 2015; Mahalingam et al., 2016). Briefly, prostate as well as vascular cells, present in a broad number of tumors, such as bladder-, breast-, hepatocellular-, renal-cancer and glioblastoma multiforme, harbor the proteolytic enzyme that is termed prostate specific membrane antigen (PSMA) in their plasma membranes. Therefore, these cells can be specifically targeted by TG-conjugates, which comprise TG in combination with oligopeptides that are substrates for PSMA. Upon cleavage of the prodrug conjugates by PSMA, TG is selectively liberated in the vicinity of the target cells, inhibits SERCA in these cells and drives them into programmed cell death. According to experiments with tumor cell lines, however, *SEC62* overexpressing tumor cells may be unresponsive or resistant against such treatment (Linxweiler et al., 2013). Therefore, murine tumor models for tumor growth or metastasis were evaluated with respect to their responsiveness to treatment with the mipsagargin/G202 analog TG in the presence of TFP, which had previously been in clinical use for the treatment of schizophrenia, and will be discussed next.

### Trifluoperazine reduces tumor growth and metastatic potential in murine tumor models

Hagerstrand et al. were the first to demonstrate induction of subcutaneous tumor growth in C. Cg/AnNTac-Foxn1^nunu^ mice, inoculated with *SEC62* overexpressing HMLE cells (Hagerstrand et al., 2013). Because of the promising *in vitro* results for TFP, *in vivo* studies in form of murine tumor models appeared to be warranted as the next logical step during recent years. One first *in vivo* study reported a significant inhibition of seeding and growth of a subcutaneously injected head and neck squamous cell carcinoma cell line (FaDu) in BALB/cAnNRj-Foxn1^nu^/Foxn1^nu^ mice by single and combined treatment with TFP and TG (Körbel et al., 2018). A second *in vivo* study focusing on lymphatic metastases addressed if the migration inhibition, which was found for various cancer cells *in vitro,* manifests as a clinically relevant phenotype in a living organism and observed a tendency of both drugs to suppress metastasis rate as well as to reduce metastasis size in an orthotopic xenograft mouse model of hypopharyngeal squamous cell carcinoma (Körner et al., 2022). Within the scope of such therapeutic concepts, the benefit of autophagy inhibitors was recently tested both at the level of gastric cancer cells and in a murine metastasis model for gastric cancer (Su et al., 2022). The striking observations were that *SEC62* overexpression affects the levels of the matrix metalloproteinases MMP2 and MMP9 plus their inhibitor TIMP-1 and promotes the migratory and invasive potential of gastric cancer cells. In addition, it was observed that autophagy blockade by hydroxychloroquine (HCQ) indeed impairs the promotive effects of *SEC62* overexpression on the migratory and invasive potential (Figure 4). Thus, it was concluded that the combination of reduction of the Sec62 level by lentiviral shRNA and autophagy blockade exerts a synergistic anti-metastatic effect and, therefore, represents a promising therapeutic strategy for metastases of gastric cancers (Su et al., 2022). However, the mechanism of TIMP-1 to MMP2 plus MMP9 balance change under conditions of *SEC62* overexpression remains to be elucidated. Based on the observation that the ER import of ADAM metalloproteinases ADAM10 and ADAM17 and the protease inhibitor SERPINE2 depends on Sec62 (Lang et al., 2022), a direct role of *SEC62* overexpression on ER import of TIMP-1 or MMP2 and MMP9 may also be considered. Furthermore, the possible influence of HCQ on ER stress tolerance remains to be analysed.

## Discussion

Against the background of stimulation of tumor metastasis and the increased tolerance to ER stress and worsened prognosis in *SEC62* overexpression, Sec62 presents itself as a potential target structure of a targeted antitumor therapy (Greiner et al., 2011a; Greiner et al., 2011b) (Figures 2, 4). Although, as previously described, several *in vitro* investigations observed that the migratory and metastatic potential of human tumor cells can significantly be reduced by a *SEC62* siRNA transfection (Greiner et al., 2011a; Linxweiler et al., 2012, 2016; Bochen et al., 2017), transfer of this technology to humans does not appear possible at present. So far, it is due to significant systemic side effects and too low concentrations at the target site that small RNA molecules have not yet succeeded in tumor therapeutics in humans. However, the possibility of a targeted *in vivo* CRISPR/Cas9-based genome editing may represent a promising future approach, as may be true for microRNAs (He et al., 2019). In search of alternative mechanisms to inhibit Sec62 function, it was shown that treating tumor cells with the CaM antagonist TFP both in terms of migratory potential and cellular calcium homeostasis achieves the same phenotype as *SEC62* silencing (Linxweiler et al., 2013) (Figure 4). However, the possible influence of TFP on pro-angiogenesis was not yet analysed. Since the CaM antagonist TFP was in clinical use for the treatment of psychiatric illnesses for many years, for this drug already exists extensive experience with a human application. Furthermore, the repurposing of this substance as a tumor therapeutic has already been developed by a few groups in recent years and has been investigated with promising results, in some cases in combination with bleomycin (Polischouk et al., 2007; Pulkoski-Gross et al., 2015; Sullivan et al., 2002; Sangodkar et al., 2012; Yeh et al., 2012; Zacharski et al., 1990; Zhelev et al., 2004). However, the possible influence of *SEC62* overexpression on the TFP effect was not yet analysed in these studies. Based on the *in vitro* data, we expect that the TFP effect may be increased by an additional treatment with an inhibitor of the SERCA, in particular, since a dose-dependent proliferation inhibition was achieved with this combination *in vitro*. Currently, several groups already deal with the application of TG or of TG-related prodrugs (such as mipsgargin) as potential tumor therapeutics in advanced solid tumor diseases (Denmeade et al., 2003, 2012). A clinical phase I study (Dose-Escalation Phase 1 Study of G-202 in patients with advanced solid tumors, Clinical Trials Government Identifier NCT01056029) showed favorable pharmacokinetic properties with acceptable toxicity in the treated patients (Mahalingam et al., 2016). Furthermore, phase II clinical trials with the prodrug mipsagargin/G202 are under way (Doan et al., 2015). Advanced investigations, and in particular clinical studies, will be required to evaluate clinical utility and response to therapy for *SEC62* overexpressing tumors with TFP and TFP plus TG-related prodrugs, which also affect angiogenesis because of the PSMA content of vascular cells (Doan et al., 2015). Along these same lines, based on the fascinating recent results on autophagy inhibitor HCQ in the metastases formation of *SEC62* overexpressing gastric tumors (Su et al., 2022), combinatorial therapies of HCQ plus TFP and HCQ plus the targeted mipsagargin/G202, respectively, should be considered as potential future therapeutic options. Notably, CQ and HCQ are used in malaria prophylaxis and therapy and discussed in the therapy of various cancer entities (reviewed by Levy et al., 2017).
